# Clinical and Analytical Validation of a Novel Urine-Based Test for the Detection of Allograft Rejection in Renal Transplant Patients

**DOI:** 10.3390/jcm9082325

**Published:** 2020-07-22

**Authors:** Niamh Nolan, Katherine Valdivieso, Rekha Mani, Joshua Y. C. Yang, Reuben D. Sarwal, Phoebe Katzenbach, Kavita Chalasani, Donna Hongo, Gladys Lugtu, Corinne Mark, Edna Chen, Reggie Nijor, David Savoca, David S. Wexler, Todd Whitson, Shih-Jwo Huang, Lucy H. Lu, Robert J. X. Zawada, Evangelos Hytopoulos, Minnie M. Sarwal

**Affiliations:** 1 NephroSant Inc., 150 North Hill Drive, Brisbane, CA 94005, USA; kvaldivieso@nephrosant.com (K.V.); rmani@nephrosant.com (R.M.); jyang@nephrosant.com (J.Y.C.Y.); rsarwal@nephrosant.com (R.D.S.); pkatzenbach@nephrosant.com (P.K.); kchalasani@nephrosant.com (K.C.); dhongo@nephrosant.com (D.H.); glugtu@nephrosant.com (G.L.); cmark@nephrosant.com (C.M.); echen@nephrosant.com (E.C.); rnijor@nephrosant.com (R.N.); dsavoca@nephrosant.com (D.S.); dwexler@nephrosant.com (D.S.W.); twhitson@nephrosant.com (T.W.); shuang@nephrosant.com (S.-J.H.); llu@nephrosant.com (L.H.L.); rzawada@nephrosant.com (R.J.X.Z.); ehpdspace@sbcglobal.net (E.H.); msarwal@nephrosant.com (M.M.S.); 2 Department of Surgery, University of California San Francisco, San Francisco, CA 94143, USA

**Keywords:** acute rejection, subclinical rejection, T cell-mediated rejection, antibody-mediated rejection, cell-free DNA, CXCL10, allograft, kidney transplant, methylated cell-free DNA, QiSant, QSant

## Abstract

In this clinical validation study, we developed and validated a urinary Q-Score generated from the quantitative test QSant, formerly known as QiSant, for the detection of biopsy-confirmed acute rejection in kidney transplants. Using a cohort of 223 distinct urine samples collected from three independent sites and from both adult and pediatric renal transplant patients, we examined the diagnostic utility of the urinary Q-Score for detection of acute rejection in renal allografts. Statistical models based upon the measurements of the six QSant biomarkers (cell-free DNA, methylated-cell-free DNA, clusterin, CXCL10, creatinine, and total protein) generated a renal transplant Q-Score that reliably differentiated stable allografts from acute rejections in both adult and pediatric renal transplant patients. The composite Q-Score was able to detect both T cell-mediated rejection and antibody-mediated rejection patients and differentiate them from stable non-rejecting patients with a receiver–operator characteristic curve area under the curve of 99.8% and an accuracy of 98.2%. Q-Scores < 32 indicated the absence of active rejection and Q-Scores ≥ 32 indicated an increased risk of active rejection. At the Q-Score cutoff of 32, the overall sensitivity was 95.8% and specificity was 99.3%. At a prevalence of 25%, positive and negative predictive values for active rejection were 98.0% and 98.6%, respectively. The Q-Score also detected subclinical rejection in patients without an elevated serum creatinine level but identified by a protocol biopsy. This study confirms that QSant is an accurate and quantitative measurement suitable for routine monitoring of renal allograft status.

## 1. Introduction

The total number of living kidney transplant recipients with a functioning graft is expected to pass 250,000 in the next 1–2 years [1]. In 2019, there were 23,401 renal transplant procedures performed, which represents a 10.6% increase from 2018 [2]. Kidney transplantation is the best treatment option for end-stage renal disease. However, the growing number of transplant allograft rejection continues to be a major problem in maintaining the value of the kidney transplant benefits. Incidence of acute rejection (AR) by one-year posttransplant among adult kidney transplant recipients is about 7.8% [1]. Scientific Registry of Transplant Recipients (SRTR) data showed that 97% of kidney transplants are working at the end of a month, 93% are working at the end of a year, and 83% are working at the end of 3 years [1]. Despite life-long immunosuppressive maintenance regimens designed to optimize the outcome, approximately 20–30% of patients experience overall renal graft failure within the first 5 years, and only 55% of transplanted kidneys last to 10 years [3].

Before molecular transplant rejection tests, such as tests to measure donor-derived cell-free DNA (dd-cfDNA) or mRNA expression profiling [4,5,6,7], became available, renal allograft injuries were commonly monitored by measuring serum creatinine (SCr) levels. Although elevated serum creatinine correlates with low estimated glomerular filtration rate (eGFR) and functional decline, SCr is an insensitive biomarker for diagnosing allograft rejection. In recent clinical validation studies of blood-based transplant rejection tests measuring dd-cfDNA, SCr has been consistently shown to be inferior to the molecular tests in identifying active rejection [5,6].

Renal biopsy is the standard in diagnosing allograft rejection. However, renal biopsy is not a convenient surveillance method for detecting rejections and is commonly used to provide a definitive confirmation for rejections. In addition to high costs, there are procedure-related risks and discomfort associated with invasive biopsies, and the histological analysis of biopsies is subjective to intra- and interobserver variations [8]. About 20% of the U.S. kidney transplant centers use a protocol-based biopsy approach to detect subclinical renal pathology. Protocol biopsy may enable early graft abnormalities detection at the stage when effective treatment can change rejection trajectory and improve long-term outcomes. However, the procedure carries a finite risk of complications and about 25% biopsies yield an inadequate specimen to determine histological rejections [9]. The disadvantages of histological evaluation for routine monitoring also include the inability to predict rejection or determine treatment response. Therefore, there is a critical clinical need for a sensitive, quantitative, non-invasive diagnostic test that can detect changes in graft status to guide clinically indicated biopsy decisions and monitor rejection risk longitudinally to improve allografts lifespan and long-term outcomes.

The QSant six biomarkers had previously been studied in the Kidney Injury Test, and the corresponding Kidney Injury Score (KIT Score) was shown to be able to assess the stage of chronic kidney disease (CKD) much earlier than proteinuria and renal function measurements alone [10]. In a foundational clinical study encompassing running 601 urine samples at the University of California San Francisco (from patients without kidney disease, with chronic kidney disease and both stable and dysfunctional kidney transplants), the same six biomarkers were adapted to generate the transplant quiescence assay called QSant, formerly known as QiSant [11], and the combination of the six biomarkers and clinical variables generated a scaled Q-Score that allowed for accurate detection of acute kidney transplant rejection and differentiated acute rejection injury from stable allografts in both children and adults [11]. 

In this study, we propose a further re-validation of the foundational clinical study [11] that developed, validated, and cross-validated the performance of the Q-Score for non-invasive diagnosis of acute rejection by the QSant assay. The primary objective of this validation study is to re-assess the performance of the QSant assay on allograft kidney rejection status, after assay transfer, assay lock, and algorithm lock in the Clinical Laboratory Improvement Amendments (CLIA) lab of NephroSant. The Q-Score scaled from 0–100 was similarly generated as discussed in the foundational clinical paper by Yang et al. [11], with the same fixed Q-Score cut-off of greater than or equal to 32 for diagnosis of acute rejection, and then applied to two separate datasets, generated in the CLIA Lab, to re-evaluate the sensitivity, specificity, negative predictive value (NPV), positive predictive value (PPV), and accuracy of assessment of allograft rejection status, as assessed by a renal allograft biopsy, paired with the urine sample processed for QSant.

The results of this study, combined with our initial foundational study by Yang et al. [11], demonstrate that the accuracy and the non-invasive nature of a QSant urine test makes it most relevant for the routine and repeated monitoring of transplant health for the lifetime of a renal allograft. The Q-Score provides detection and quantitative evaluation of rejection injury, which can be tracked over time to influence choices/dose of immunosuppression. An accurate measure of changes in kidney transplant health over time can inform clinical decision-making and support choices of treatment regimens. Increasing use and applications of the Q-Score in prospective clinical trials may further support the Q-Score as a surrogate endpoint for acute rejection therapy effectiveness and kidney transplant rejection outcome measurements without the need of repeated invasive transplant biopsies.

## 2. Experimental Section 

### 2.1. Study Population and Sample Collection

The urine samples used in the clinical validation study were prospectively collected between 2010 and 2018 from adult (18 to 76 years of age) and pediatric (3 to 18 years of age) kidney transplant recipients who had transplant surgeries at the Stanford University Medical Center, the University of California San Francisco, or the Instituto Nacional de Ciencias Medicas y Nutricion in Mexico. 

The studies were approved by the institutional review boards (IRBs) of all three institutions. Stanford University and Institute Nacional de Ciencias Medicas y Nutricion samples were obtained through a materials transfer agreement. The relevant University of California San Francisco IRB for this research was IRB #14-13573, with the most recent reapproval date of 29 June 2020. Informed consent for participation in the research was obtained from all patients or their legal guardians. The study protocol conforms to the ethical guidelines of the 1975 Declaration of Helsinki. The clinical and research activities being reported are consistent with the Principles of the Declaration of Istanbul, as outlined in the Declaration of Istanbul on Organ Trafficking and Transplant Tourism.

Urine samples were collected before the performance of an indicated or protocol biopsy in sterile containers with a pre-defined collection protocol. On arrival to the lab, urine samples were centrifuged at 2000× *g* at 4 °C for 30 min. The supernatant was aliquoted, and pH was adjusted to 7.0 by adding 1 part 1 M tris-HCl to 10 parts urine supernatant. The urine was stored at −80 °C until it was ready to use. 

### 2.2. Kidney Biopsy

All samples were biopsy-matched and had urine collected at the time of clinical dysfunction, for-cause biopsy, or protocol biopsy. All kidney biopsies were analyzed by a pathologist who was blinded to the clinical course and graded using the 2017 Banff classification [1]. Intragraft C4d stains were performed to assess for antibody-mediated rejection (ABMR). AR was defined, at minimum, by the following criteria: (1) T-cell-mediated rejection (TCMR) consisting of either a tubulitis (t) score > 2 accompanied by an interstitial inflammation (i) score > 2 or vascular changes (v) score > 0; (2) C4d-positive ABMR consisting of positive donor-specific antibodies (DSAs) (MFI > 1500) with a glomerulitis (g) score > 0 or peritubular capillaritis score (ptc) > 0 or v > 0 with unexplained acute tubular necrosis/thrombotic microangiopathy (ATN/TMA) with C4d = 2; or (3) C4d-negative ABMR consisting of positive DSA with unexplained ATN/TMA with g + ptc ≥ 2 and C4d = 0 or 1. Subclinical AR histology on a surveillance biopsy is similar to acute rejection. Stable transplant allografts (STAs) were defined by an absence of substantial injury on the matched biopsy pathology and definitions of the inflammation or i score and the tubulitis or t score. 

### 2.3. QSant Assay

The QSant assay measures six urinary biomarkers including cfDNA, methylated cfDNA (m-cfDNA), CXCL10, clusterin, creatinine, and total protein with measurements performed as previously described [10,11]. Briefly, the cfDNA was measured using a proprietary biotinylated probe complementary to the Arthrobacter luteus (ALU) human element [11] and streptavidin–horseradish peroxidase (HRP) (R&D Systems). Custom generated ELISAs for m-cfDNA, CXCL10, and clusterin concentration were used for these biomarkers. Both DNA assays used SuperSignal ELISA Femto Substrate (Thermo Fisher Scientific) for luminescent detection. FDA-approved tests on the Beckman Coulter AU400 analyzer were used for the measurements of creatinine and total protein. All biomarker assay and measurement interpolation calculations were performed blinded to the clinical information.

### 2.4. Statistical Analysis and Algorithm Development

The primary objective of this analysis was to assess the performance of these six urine-based biomarkers to inform on allograft kidney rejection status. The study involved 223 kidney transplant urine samples collected from 215 pediatric and adult recipients who had undergone transplant surgery at three different centers. Out of the 223 samples, acute kidney allograft rejection was observed in 71 samples, while 152 samples displayed stable allografts. Additionally, urine samples were collected from these patients from 1 to 1539 days post-transplant.

Random sampling was used to split the 223 samples into a training (*n* = 157 with 45 AR cases and 112 STA controls) set and a validation set (*n* = 66 with 26 AR cases and 40 STA controls). Sample phenotypes were based on the pathology of the paired kidney transplant biopsy, utilizing the Banff pathology classification [12]. Non-parametric Wilcoxon Mann–Whitney test and chi-squared test were used to test the significant difference between training and test cohorts for demographic variables. Random forest model based on the measurements of the six biomarkers and days post-transplant was built to predict kidney injury status. Additionally, the Q-Score obtained using the random forest model was used for predicting allograft kidney rejection. To evaluate the performance of the model, we used area under the curve (AUC), sensitivity, specificity to discriminate the acute kidney rejection group from the non-rejection group. Methods such as fivefold cross validation and bootstrapping were used to assess the performance of the random forest model. All the above analysis was performed with the use of R 1.2.5 or Python 3.7.0. Visualizations were performed in GraphPad Prism 8.4.3 (GraphPad, Carlsbad, CA, USA).

## 3. Results

### 3.1. Study Design and Cohort Description

**Figure 1:**
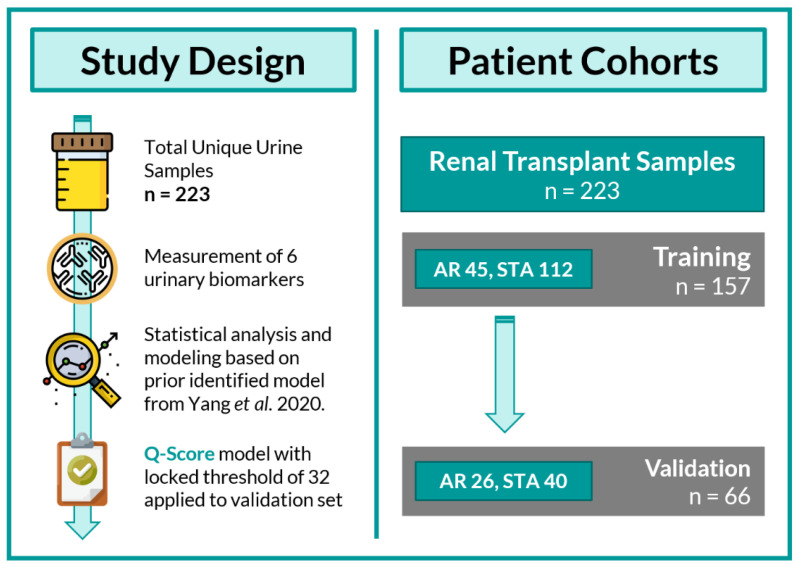
Study design: This study involved a total of 223 urine samples. Each urine sample was paired with renal transplant biopsy for phenotype classification into either of the following diagnoses: stable/healthy (STA: *n* = 152) and acute rejection (AR: *n* = 71). The urinary biomarkers were measured on all the urine samples collected, and statistical analyses were performed on stable and acute rejection transplant patients. Samples were randomly split into training (*n* = 157) and validation sets (*n* = 66). The Q-Score model was developed using training data and then applied to the validation set.

In this clinical validation study, we employed optimized CLIA-validated assays to measure the six biomarkers (cfDNA, m-cfDNA, clusterin, CXCL10, creatinine, and total protein) in a kidney transplant cohort comprised of 71 AR and 152 of STA to train and validate the QSant test using the same statistical and modeling approach as that in the prior clinical study (Figure 1).

**Figure 2:**
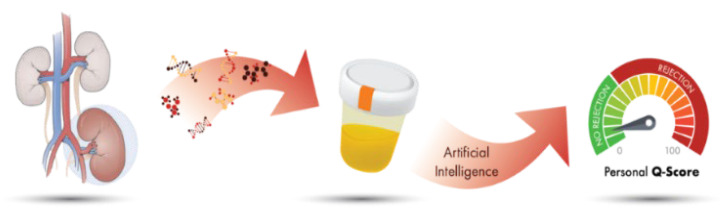
The QSant test is designed to quantitatively measure acute kidney transplant rejection using patient urine samples. The test measures six urinary biomarkers: cell-free DNA (cfDNA), methylated cfDNA (m-cfDNA), clusterin, CXCL10, creatinine, and total protein in renal transplant patients. The six-biomarker data are integrated into an algorithm to calculate a composite kidney rejection risk score, Q-Score, from a scale of 0–100 to reflect the probability of allograft rejection risk.

The study was designed to demonstrate that the Q-Score algorithm can (a) differentiate AR from STA better than the standard of care test serum creatinine (SCr), (b) detect different rejection phonotypes (TCMR and ABMR), (c) detect AR in both adult and pediatric transplant patients, (d) detect AR regardless the time elapsed since transplantation, and (e) detect subclinical AR. This study cohort is comprised of 194 distinct adult and 29 pediatric kidney transplant recipient urine samples with matched biopsy as a reference standard where the urine was collected immediately before the performance of a biopsy (Figure 2). We assessed the performance of the QSant assays across a wide range of patient characteristics, such as recipient age (age range of 3–76 years), genders (51.6% males and 48.4% females), recipient/donor ethnicities, causes of end-stage renal disease (ESRD), and donor sources (Table 1).

### 3.2. Differentiation of AR Patients from STA Patients

**Figure 3:**
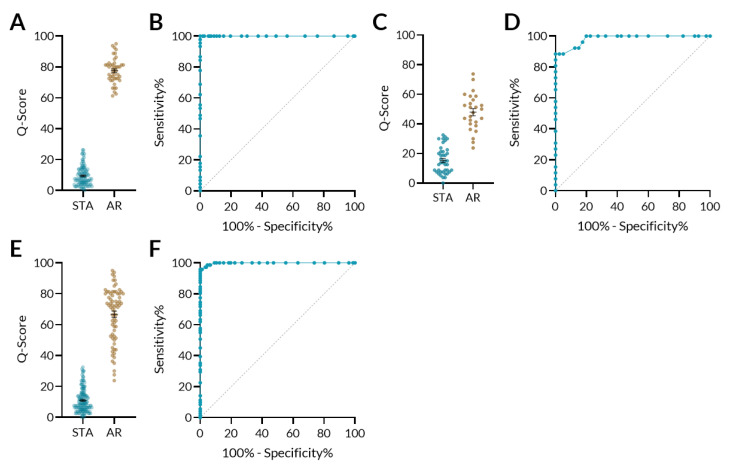
QSant performance: The selected urinary biomarkers could segregate non-rejection patients from those with acute rejection. (**A**) The model was trained on 157 samples consisting of 112 stable transplant allograft (STA) and 45 acute rejection (AR) samples in the training set to generate a scaled score ranging from 0 to 100. At a threshold of 32, the sensitivity was 100% and the specificity was 100%. (**B**) The area under the curve (AUC) of the receiver–operator characteristic (ROC) curve of the training cohort was 100%. (**C**) In the independent validation set, the Q-Score model was applied to a set of 66 independent samples consisting of 40 STA and 26 AR samples. At the pre-determined threshold of 32, the sensitivity was 95.8% (95% CI: 78.9–99.8%) and the specificity was 92.9% (95% CI: 80.5%-98.5%). (**D**) The AUC of the ROC curve of the validation cohort was 98.3% (95% CI: 0.96–1.0). (**E**) At the threshold of 32 for the entire dataset, sensitivity was 95.8% (95% CI: 88.14–99.12%) and specificity was 99.3% (95% CI: 96.4–99.9%). The PPV was 98.0% (95% CI: 87.3–99.7%) and the NPV was 98.6% (95% CI: 95.9–99.5%). (F) AUC for the entire data was 99.8%.

The six urinary biomarkers have been demonstrated to differentiate AR from stable renal transplants [11]. In this study, the Q-Score algorithm was optimized on the basis of the data generated using a random forest bootstrap model on 157 samples in the training set. An optimal threshold of 32 was established in the training data to separate Q-Score into STA (<32) and acute rejection (≥32) groups. The Q-Score in discriminating stable (STA) and AR outcomes was further validated on 66 samples in the validation set. We used AUC to evaluate the performance of the predictive model. A receiver–operator characteristic (ROC) curve of the urine score had an area under the curve (AUC) of 100% in the training set (Figure 3B). Applying the model to the validation set, we obtained AUC of 98.3% (95% CI: 0.96–1.0) (Figure 3D). In both training and validation sets, Q-Score clearly distinguished STA from AR patients (Figure 3A,C). At the defined threshold of 32, the sensitivity and specificity were both 100% in the training set. In the validation set, sensitivity and specificity were 95.8% and 92.9%, respectively.

### 3.3. Comparison of QSant Performance to Clinical Parameters

**Figure 4:**
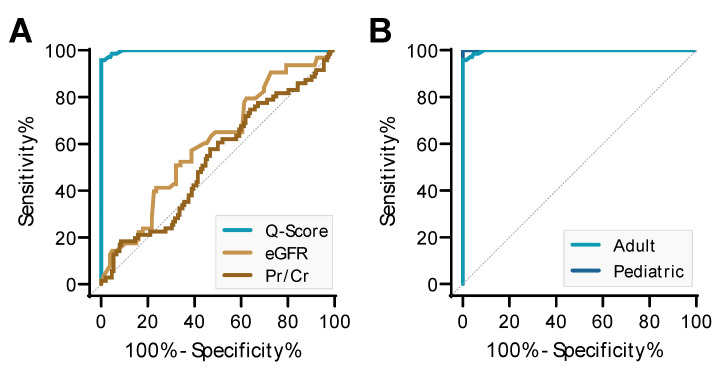
Q-Score performance compared to serum creatinine (SCr) and the estimated glomerular filtration rate (eGFR) and by recipient age. (**A**) The Q-Score showed superior performance compared with SCr and eGFR in discriminating AR from STA. (**B**) The performance of the Q-Score in different transplant patient age groups to differentiate AR from STA. Q-Score performed equally well in diagnosing pediatric transplant recipients (10 months to 18 years old) and in adult transplant recipients >18 years old. The AR prediction AUC of the ROC for pediatric patients was 100% (95% CI: 1.000–1.000; STA 25, AR 4) and the AUC of the ROC for adult patients was 99.8% (95% CI: 0.995–1.000; STA 127, AR 67).

The clinical performance of Q-Score in differentiating AR and stable transplants had an AUC of 99.8% (95% CI: 0.995-1.0), which is much better that of SCr, which had an AUC of 53.2% (95% CI: 0.45–0.61), and eGFR, which had an AUC of 59.7% (95% CI: 0.51–0.69) (Figure 4A). 

### 3.4. QSant Utility in Pediatric Transplant Patients

QSant Q-Score was able to detect AR irrespective of recipient age (Figure 4B). The AUC for discriminating STA from AR in pediatric transplant patients was 100% (95% CI: 1.000 to 1.000; *p* < 0.0001) and 99.8% in adult transplant patients (95% CI: 0.995 to 1.000; *p* < 0.0001).

### 3.5. Correlations of Q-Scores to Different Transplant Rejection Phenotypes

**Figure 5:**
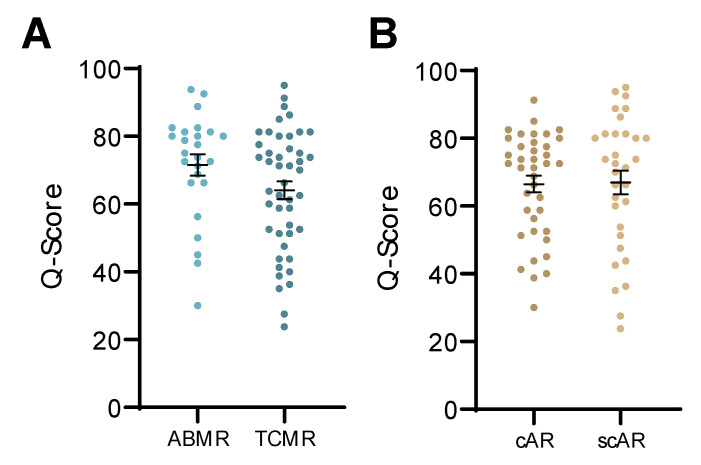
The QSant assay can detect different clinical transplant rejection phenotypes and can detect both clinical and sub-clinical acute rejection. (**A**) The study composed of AR patients with antibody-mediated rejection (ABMR; *n* = 24) and T-cell-mediated rejection (TCMR; *n* = 44). No statistically significant difference was observed between two groups, which indicated that Q-Score can effectively detect both rejection phenotypes (*p* = 0.076). (**B**) The Q-Score was not significantly different between clinical rejection (*n* = 39) and sub-clinical rejection (*n* = 32) groups (*p* = 0.720).

In this study, there were a total of 68 biopsy-confirmed rejections that met Banff histological criteria for classifying a rejection either as TCMR (*n* = 44) or ABMR (*n* = 24). Q-Scores were equally efficient in identifying both TCMR and ABMR rejections in this cohort (Figure 5A).

### 3.6. Detection of Subclinical Allograft Rejection

We evaluated the QSant assay in patients with protocol and for-cause biopsies (*n* = 223) at 1 (8%), 3 (24%), 6 (16%), and 12 (11%) months after renal transplant, and when clinically indicated. Of the set of urine samples with AR (*n* = 71), we were able to assess the performance of Q-Score on detecting subclinical AR (scAR; *n* =32) diagnosed at the time of protocol biopsies, when there was stable graft function, compared to clinical AR (cAR; *n* = 39) (Figure 5B). 

### 3.7. Timing of QSant Post-Transplantation

**Figure 6:**
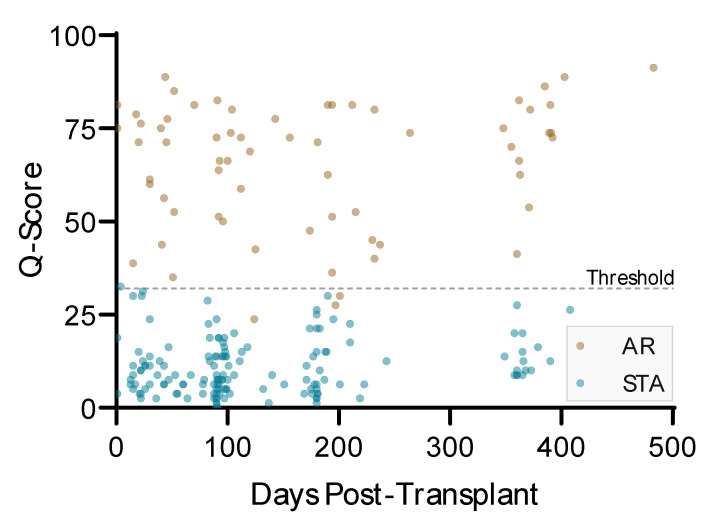
QSant can detect AR regardless of the time of development of acute rejection. The graph illustrates the Q-Score of each study patient and time from transplantation date to biopsy/sample collection date. Eight points beyond 500 days post-transplant are not shown in the graph. The data show that the QSant test can be used effectively in renal transplant patients 1 day after the procedure without the need to wait for the individual biomarkers to fall to their individual post-transplant stable steady-state levels post-transplantation.

QSant test performed equally well in renal recipients from 1 day after transplant to 4 years post-transplant date. It has been widely reported that blood levels of dd-cfDNA increased immediately post-transplant and rapidly fell to steady-state baseline levels in uncomplicated patients by around 7 to 10 days post-transplant [9]. Although the individual urine biomarkers can measure variations in levels early post-transplant, the sensitivity and specificity of the Q-Score to diagnose acute rejection is not impacted by proximity to the transplantation procedure (Figure 6).

### 3.8. Avoiding Unnecessary Biopsies

**Figure 7:**
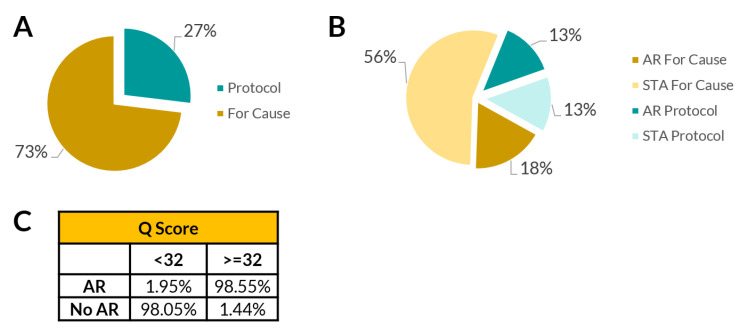
The urine QSant assay and Q-Score can help reduce unnecessary renal biopsies. (**A**) The study cohort contains a total of 163 for-cause biopsies, of which 124 had Q-Scores below the rejection threshold of 32. Of the 60 protocol biopsies, 30 had a score below the rejection threshold of 32. The breakdown of different biopsy diagnoses paired with the sample is shown in the figure as percentages. (**B**) In this study cohort, if physicians had Q-Scores on hand, the test results would have impacted their decision to not order biopsy for 69% (154/223) of the time. (**C**) Accuracy of biopsy (for-cause vs. protocol) and the Q-Score (score < 32 vs. score ≥ 32) for classifying AR and STA using Banff classification confirmed biopsies as reference standard.

Out of 223 total samples, there were 60 patients who underwent protocol biopsies and 163 patients who underwent for-cause biopsies (Figure 7A,B). On the basis of Q-Score, 44% of patients were identified as healthy but still took protocol biopsy. Additionally, 25% patients who took for-cause biopsy were identified as healthy. The accuracy table for biopsy (for-cause vs. protocol) and the Q-Score (score <32 vs. >32) for AR and no AR classification shows the utility of the Q-Score (Figure 7C).

## 4. Discussion

Renal transplant recipients generally require life-long immunosuppression to prevent graft rejection. However, despite improved immunosuppressive maintenance regimens designed to optimize the outcome, approximately 20–30% of patients experience overall renal graft failure within the first 5 years, and only 55% of transplanted kidneys last to 10 years [3]. Our goal w to create a safe and high-performance clinical test that can be used at sufficient frequency to detect early stage graft dysfunctions where intervention can alter the progression course and extend allograft lifespan. 

In previous clinical studies [10,11], the six selected urinary biomarkers were able to effectively measure the burden of chronic kidney injury and detect acute renal allograft rejection with much higher sensitivity and specificity than eGFR, protein/creatinine, or SCr. The focus of this paper was to refine on the basis of optimized assays and validate the clinical performance of Q-Score for the accurate detection of acute allograft rejection as defined by Banff classification criteria using biopsy-matched clinical samples and optimized CLIA-validated assays.

Our validation study results confirmed our previous findings that the QSant test enables objective and early clinical assessment of renal allograft [11]. The biomarker panel is robust and has superior performance when compared with existing tests for detecting risk for AR in all ages of renal transplant patients. Q-Score quiescent threshold also provides additional clinically relevant information.

In a large serial protocol biopsy study, renal transplant protocol biopsies were shown to have a 1% rate of causing major complications, as well as having other less severe complications, such as 3.5% risk of gross hematuria and 2.5% risk of perirenal hematomas [13]. QSant can effectively reduce the need to implement a screening protocol biopsy to monitor subclinical allograft rejections. This study, similar to the foundation study by Yang et al. [11], shows that the majority of protocol biopsies done to screen for acute rejection could be avoided; 65% were unnecessary in the Yang et al. [11] study and 73% of protocol biopsies could have been avoided in the current study cohort, as all these patients had a Q-Score below the 32 (rejection) threshold. Our study data suggested that the inclusion of a high-performance biomarker test can effectively reduce the use of for-cause and surveillance biopsies by 69%.

AR covers a wide range of complex syndromes and underlying etiologies. However, the Q-Score quiescent threshold was able to effectively detect both TCMR and ABMR in the study cohort, showing that the two pathologies were not distinctly different from one another. At this time, the QSant assay cannot discriminate between ABMR and TCMR. While ABMR manifests with microcirculation damage and DSA, TCMR manifests with interstitial–epithelial changes [2]. These differences are clinically meaningful because the treatment for each type of rejection is different For TCMR, pulse high-dose intravenous glucocorticoids and rabbit anti-thymocyte globulin (rATG/Thymoglobulin) are treatments of choice [3]. For ABMR, a combination of glucocorticoids, plasmapheresis, and intravenous immune globulin is used [4]. We imagine that the QSant assay would be used to indicate if an episode of acute rejection is occurring, at which time a biopsy could be ordered to determine the exact type of acute rejection.

In addition, QSant can be safely performed 1 day post transplantation and is applicable to detect early risk of acute rejection. The inclusion of Q-Score information obtained from QSant assays in clinical management can allow early and proactive use of immunosuppressive therapy while titrating the drug levels to suit each patient’s need.

Ultimately the integration of objective molecular testing into the existing histopathology criteria would create a true gold standard for allograft rejection diagnosis that is robust and has strong histological rationale. QSant can add real value to the healthcare system by reducing unnecessary biopsies with accurate diagnosis of acute rejection, irrespective of a change in the serum creatinine. The high sensitivity of the QSant urine test can avoid the risk of an invasive biopsy when a rise in serum creatinine is due to a cause other than acute rejection, and the high specificity of the urine QSant test can avoid an unnecessary protocol biopsy by identifying patients who have normal serum creatinine and no rejection.

The main limitation of the study was that even though we used one pathologist to conduct centralized pathohistological lesion grading to minimize observer variations, this gold standard’s innate sampling limitations is known to have a false negative rate up to 30% in detecting rejections. The cohort had a single urine collection from the subclinical disease patients. Had we had subsequent biopsy confirmation data of STA patients with initial high Q-Score in this study, we could potentially reclassify these STA patients as scAR instead of false-positive AR. Another limitation of this cross-sectional study was not having longitudinal samples to demonstrate the progression or improvement of Q-Scores to reflect the impact of test-guided treatment on immune rejection risk and outcomes. 

## 5. Conclusions

In the validation cohort, the levels of six urinary biomarkers—cfDNA, m-cfDNA, CXCL-10, clusterin, total protein, and creatinine—along with days post-transplant were used to generate a composite Q-Score. The study demonstrated multiple benefits in aiding the management of renal transplant patients: (i) the overall benefit in detecting clinical allograft rejection without performing unnecessary biopsies, (ii) the net reduction in unnecessary biopsies without missing any of the clinical rejections, (iii) detection of TCMR and ABMR with equally high accuracy, (iv) detection of subclinical rejection with high accuracy, and (v) applicability to all ages (3 to 76 years) of transplantation patients.

The QSant test is intended to supplement evaluation and management of kidney injury and acute rejection in patients who have undergone renal transplantation, regardless of recipient age or whether the patient is the recipient of a repeat or multi-organ transplant. It can be used by physicians considering the diagnosis of acute rejection, helping to rule in or out this condition. In conclusion, the QSant assay is a multimodal risk assessment approach that can improve the diagnosis and management of renal transplant patients and reduce unnecessary biopsy.

## Figures and Tables

**Table 1:** Baseline characteristics of study patients. ^a^

Phenotype Characteristic	Training (157 Samples)	Validation (66 Samples)	Overall (223 Samples)	*p*-Value
Recipient				
• Recipient age, year (SD) (min, max)	32.6 (14.8) (3, 76)	31.7 (13.3) (4, 70)	32.4 (14.4) (3, 76)	0.138
• Recipient gender, female (%)	76 (48.4%)	32 (48.5%)	108 (48.4%)	1.000
Donor Source				
• Deceased donor (%)	50 (31.8%)	15 (22.7%)	65 (29.1%)	0.106
• Living related (%)	80 (51.0%)	32 (48.5%)	112 (50.2%)	
• Living unrelated (%)	25 (15.9%)	19 (28.8%)	44 (19.7%)	
• Unspecified (%)	2 (1.3%)	0 (0.0%)	2 (0.9%)	
Cause of ESRD				
• Congenital	50 (31.8%)	24 (36.4%)	74 (33.2%)	0.854
• Diabetes mellitus	16 (10.2%)	3 (4.5%)	19 (8.5%)	
• Glomerulonephritis	10 (6.4%)	4 (6.1%)	14 (6.3%)	
• Hypertension	14 (8.9%)	6 (9.1%)	20 (9.0%)	
• Immune-mediated	28 (17.8%)	14 (21.2%)	42 (18.8%)	
• Obstructive	8 (5.1%)	4 (6.1%)	12 (5.4%)	
• Other	29 (18.5%)	11 (16.7%)	40 (17.9%)	
• Unspecified	2 (1.3%)	0 (0.0%)	2 (0.9%)	
Ethnicity				
• African American	5 (3.2%)	2 (3.0%)	7 (3.1%)	0.634
• Asian	4 (2.5%)	2 (3.0%)	6 (2.7%)	
• Caucasian	5 (3.2%)	4 (6.1%)	9 (4.0%)	
• Hispanic	105 (66.9%)	48 (72.7%)	153 (68.6%)	
• Other	2 (1.3%)	0 (0.0%)	2 (0.9%)	
• Unspecified	36 (22.9%)	10 (15.2%)	46 (20.6%)	
Phenotype				
• AR	45 (28.7%)	26 (39.4%)	71 (31.8%)	0.158
• STA	112 (71.3%)	40 (60.6%)	152 (68.2%)	

^a^ Values are reported in the given units with standard deviation or proportion in parentheses. Characteristics and demographic information of recipients is based on the day of urine collection. No statistically significant differences were observed between the training and test cohorts.
